# Disturbed lipid and amino acid metabolisms in COVID-19 patients

**DOI:** 10.1007/s00109-022-02177-4

**Published:** 2022-01-22

**Authors:** Mojgan Masoodi, Manuela Peschka, Stefan Schmiedel, Munif Haddad, Maike Frye, Coen Maas, Ansgar Lohse, Samuel Huber, Paulus Kirchhof, Jerzy-Roch Nofer, Thomas Renné

**Affiliations:** 1 Institute of Clinical Chemistry, Inselspital, Bern University Hospital, Bern, Switzerland; 2 Institute of Clinical Chemistry and Laboratory Medicine, University Medical Center Hamburg-Eppendorf, Martinistr. 52, D-20251 Hamburg, Germany; 3 Department of Clinical Chemistry and Haematology, University Medical Center Utrecht, Utrecht, University, Utrecht, the Netherlands; 4 Center for Internal Medicine, Clinic of Gastroenterology, Infectiology and Tropical Medicine, University Medical Center Hamburg-Eppendorf, Hamburg, Germany; 5 Department of Cardiology, University Heart and Vascular Center UKE Hamburg, University Medical Center Hamburg-Eppendorf, Hamburg, Germany; 6 Institute of Cardiovascular Sciences, University of Birmingham, Birmingham, UK; 7 German Center for Cardiovascular Research (DZHK), Partner site Hamburg/Kiel/Lubeck, Hamburg, Germany; 8 Central Laboratory Facility, University Hospital Münster, Münster, Germany; 9 Irish Centre for Vascular Biology, School of Pharmacy and Biomolecular Sciences, Royal College of Surgeons in Ireland, Dublin, Ireland; 10 Center for Thrombosis and Hemostasis (CTH), Johannes Gutenberg University Medical Center, Mainz, Germany

## Abstract

The Coronavirus disease 2019 (COVID-19) pandemic is overwhelming the healthcare systems. Identification of systemic reactions underlying COVID-19 will lead to new biomarkers and therapeutic targets for monitoring and early intervention in this viral infection. We performed targeted metabolomics covering up to 630 metabolites within several key metabolic pathways in plasma samples of 20 hospitalized COVID-19 patients and 37 matched controls. Plasma metabolic signatures specifically differentiated severe COVID-19 from control patients. The identified metabolic signatures indicated distinct alterations in both lipid and amino acid metabolisms in COVID-19 compared to control patient plasma. Systems biology-based analyses identified sphingolipid, tryptophan, tyrosine, glutamine, arginine, and arachidonic acid metabolism as mostly impacted pathways in COVID-19 patients. Notably, gamma-aminobutyric acid (GABA) was significantly reduced in COVID-19 patients and GABA plasma levels allowed for stratification of COVID-19 patients with high sensitivity and specificity. The data reveal large metabolic disturbances in COVID-19 patients and suggest use of GABA as potential biomarker and therapeutic target for the infection.

**Supplementary information:**

The online version contains supplementary material available at 10.1007/s00109-022-02177-4.

## Background

Coronavirus disease 2019 (COVID-19) patients suffer from severe acute respiratory syndrome coronavirus 2 (SARS-CoV-2) infection that often results in respiratory tract infection associated with fever, persistent dry cough, chills, muscle pain, headaches, and diarrhea [1, 2]. The current pandemic creates severe strains on healthcare systems worldwide due to a relatively small proportion of infected patients who deteriorate into a severe respiratory distress syndrome 7–10 days after the first symptoms [3]. Pneumonia is accompanied by a “cytokine storm” that is characterized by sustained elevated interleukin-6 (IL)-6, activated endothelium, increased angiotensin-converting enzyme 2, and severe thromboembolic complications [4–6]. The only therapy that has been shown to improve outcomes in patients with severe SARS-CoV-2 infection is dexamethasone [7], demonstrating that understanding and countering the systemic reaction to severe SARS-CoV-2 infection has the potential to identify new therapeutic targets to improve outcomes of these severely ill patients. However, despite the urgency and global health implications, the metabolic characteristics of patients with COVID-19 are not well understood.

Several clinical parameters have been found to predict severe SARS-CoV-2 infection and worse outcomes, including male sex, pre-existing metabolic disorders, and comorbidities such as hypertension, diabetes, cardiovascular disease, and respiratory diseases [8, 9]. The association between obesity, diabetes, and older age with unfavorable clinical outcome suggests that metabolic disturbances may play important roles in COVID-19 patients. Furthermore, clinical data suggest that SARS-CoV-2 infection aggravates diabetic ketoacidosis [10], liver dysfunction [1, 11], and might trigger metabolic disorder. Some biomarkers to monitor disease state assess severity of COVID-19 patients have been identified including IL-6, C-reactive protein (CRP), alanine aminotransferase (ALT), aspartate aminotransferase (AST) [11], plasma D-dimers [12] as well as leukocyte and lymphocyte counts [13]. Furthermore, an increase in inflammatory cytokines and chemokines such as tumor necrosis factor alpha (TNF-α), interferon-γ-induced protein 10 (IP-10), monocyte chemoattractant protein 1 (MCP-1), chemokine (C–C motif), ligand 3 (CCL-3), and interleukins (IL-2, IL-6, IL-7, IL-10) [1] is strongly associated with COVID-19 disease. Although readily accessible in clinical practice, these parameters are hardly specific for COVID-19 [14] and specific predictive biomarkers that originate from a disturbed molecular machinery of SARS-CoV-2-infected cells are eagerly required. In addition, the identification of altered metabolic pathways could facilitate tailored treatment and disease prevention, leading to improved strategies for personalized medicine for this highly contagious and infectious disease. Plasma metabolomics allows for detection of hundreds of small molecules and can provide a metabolic signature that reflects the metabolic status of distinct organs due to the exchange of metabolites between tissues and plasma. Mass spectrometry-based metabolomics and lipidomics are established approaches to identify disease-specific metabolic profiles for diagnosis in personalized medicine [15].

Here, we performed a cross-sectional study comparing the metabolic profile of 20 hospitalized patients with severe SARS-CoV-2 infection and 37 matched hospitalized control patients without SARS-CoV-2. We demonstrate a distinct metabolomic pattern in COVID-19 patients that clearly discriminates COVID-19 and other patients. Pathway analysis identified metabolisms of lipids and biogenic amine to be affected by COVID-19. Among other novel findings, we found reduced gamma-aminobutyric acid (GABA) plasma concentrations in COVID-19 patients, which was inversely associated with inflammatory status. Our findings define metabolic alterations in SARS-CoV-2-infected individuals and shed light on pathophysiological processes that contribute to COVID-19 with potential diagnostic and therapeutic indications. The GABA pathway emerges as a potential target pathway modulating and monitoring outcomes in SARS-CoV-2 infection.

## Methods

### Study population and blood collection

The study included 20 patients with SARS-CoV-2 infection diagnosed by polymerase chain reaction (PCR) of nasopharyngeal swabs and 37 SARS-CoV-2-negative control patients both hospitalized at the University Clinical Center Eppendorf in April and May 2020. SARS-CoV-2 patients and controls were matched for sex, age, and comorbidities. COVID-19 patients generally suffer from primary diseases and comorbidities. To peak out metabolic changes which can be ascribed COVID-19, these comorbidities were matched. Only diabetes mellitus remained higher in the COVID-19 cohort (Table 1) and will be discussed later. All control patients had no obvious infections and CRP levels below 6 mg/L. Both COVID-19 and control patients received similar standard hospital meals at regular day times. EDTA blood samples were obtained in the morning, centrifuged immediately at 2.500 × g for 10 min, aliquoted and stored at -80 °C until analysis.

### Clinical chemistry analysis

Analysis of patient samples was performed at the Institute for Clinical Chemistry and Laboratory Medicine, University Clinical Center Eppendorf. Concentrations of IL-6 were determined using electro-chemiluminescence immunoassay (ECLIA, Roche, Mannheim, Germany) on a Cobas e411 automated analyzer. Sodium, potassium, activities of ALT & AST, and concentrations of bilirubin, creatinine, blood urea nitrogen, cholesterol, triglycerides, HDL-cholesterol, CRP, and apolipoprotein A-I (apo A-I) were determined on a Attelica Solution (Siemens Healthineers, Eschborn, Germany) as described by the manufacturer. Blood counts were determined on Advia 2120 hematology analyzers (Siemens). SARS-Cov-2 infection status was determined using a quantitative PCR assay.

### Targeted metabolomic analysis by UPLC-MS/MS

Recently, sera of 46 Chinese COVID-19 patients have been analyzed by targeted proteomics and metabolomics [16]. To compare the metabolic signature in European SARS-CoV-2 patients, we used ultrahigh pressure liquid chromatography-tandem mass spectrometry (UPLC-MS/MS) to acquire data in both positive and negative ionization modes that allowed for identification and quantification of 630 metabolites. Plasma samples were processed using the MxP® Quant 500 Kit (BIOCRATES Life Sciences AG, Innsbruck, Austria) according to manufacturer instructions. Briefly, 10 µL of each EDTA plasma sample, calibration standard and control sample were transferred onto a filter containing internal standards for internal standard normalization. Filters were dried under a stream of nitrogen using a pressure manifold (Waters, Eschborn, Germany). Samples were incubated with derivatization reagent phenyl isocyanate for 60 min. After drying under nitrogen, analytes were extracted with 5 mmol/L ammonium acetate in methanol and the eluate was further diluted for the ultrahigh pressure liquid chromatographic-tandem mass spectrometry (UPLC-MS/MS) analysis. The targeted analysis covered 630 metabolites (https://biocrates.com/mxp-quant-500-kit/) detected by MS/MS after UPLC separation and flow injection analysis (FIA). Each measurement required two UPLC runs and three FIA runs to cover all metabolites. All analyses were performed on an ACQUITY UPLC I-Class system (Waters) coupled to a Xevo TQ-S mass spectrometer (Waters). Reversed phase chromatographic separation was accomplished using a C18 LC-column (BIOCRATES) with 0.2% formic acid in water 0.2% formic acid in acetonitrile as eluent system. FIA solvent was methanol with a modifier, which was provided by the kit manufacturer. Metabolite quantification was performed based on a seven-point curve or one-point calibration and internal standard normalization. The obtained raw data are provided as a supplemental file.

### Statistical methods and pathway analysis

431 metabolites out of 630 passed the quality control check and were used for statistical analysis. Metabolites with more than 40% missing values were excluded during statistical analysis and the remaining missing values were imputed based on ${1}/_{5}$ of the lowest value. Wilcoxon Rank Sum Test was performed, and FDR correction was applied using Benjamini–Hochberg correction. Log2 fold-change (log2 FC) was calculated with adjusted p values. We identified 72 metabolites that were significantly different between the two groups as shown in Suppl. Table 1. Subsequently, we performed hierarchical cluster analysis using ward clustering. A scaled principal component analysis (PCA) was performed using the prcomp R package on the 72 significantly associated metabolites listed in Suppl. Table 1. The two first principal component axes account for 44% of the variance and presents the separation between SARS-Cov-2 and control subjects (Suppl. Fig. 1). The ROC analysis was performed based on the Wilcoxon test on the entire analysis set. The predicted outcome was the COVID-19 status. The area under the ROC curve (AUC) and its 95% confidence interval were computed with the pROC R package and compared with the IL-6 ROC profile using the DeLong method (Suppl. Fig. 2 and Suppl. Table 4).

Statistical analysis and pathway analysis was performed using an in-house developed R-based package (version 3.5.2). The statistical analysis of clinical data and standard laboratory parameters was performed using χ2 test, Student t-test, or Welch test. Parameters with non-Gaussian distribution as determined with Smirnov–Kolmogorov test, underwent logarithmic normalization before analysis. Analysis of low and high IL-6 subgroups was accomplished with ANOVA with Neumann–Student–Keul *post-hoc* test, or Kruskal–Wallis test with Conover post-hoc test for normally and non-normally distributed parameters, respectively. Pathway analysis was performed on statistically different metabolites using an in-house developed software. We used the hypergeometric test for the over-representation analysis and calculation of p-values and the relative-between centrality measures for pathway topology analysis and calculation of Pathway Impact.

### Ethical statement

This observational study was conducted according to the Declaration of Helsinki, in accordance with good clinical practice guidelines, and approved by the University Clinical Center Eppendorf Review Board. Residual samples were obtained following the completion of routine clinical laboratory testing for each subject and anonymized before evaluation for research purposes.

## Results

### Characterization of study patients

The SARS-CoV-2 patients presented with moderate symptoms. At the time of blood sampling, patients did not receive intensive care treatment and all enrolled patients survived the infection. The SARS-CoV-2-negative patients were hospitalized for cardiovascular diseases (16/37, 43%), for cancer (7/37, 19%), for trauma (6/37, 16%), for kidney diseases (3/37, 8%), for lung (2/37, 5%) and psychiatric disorders (2/37, 5%), and a single individual for general diagnostic workup (1/37, 3%). Cohorts were matched for gender and age (Table 1). Prevalence of diabetes was higher in SARS-CoV-2-positive patients, while other comorbidities did not differ between groups. Inflammatory biomarkers, such as IL-6 and CRP, are predictive biomarkers in COVID-19 patients [17] and were significantly increased in the SARS-CoV-2-positive group compared to the control group. Table 1 summarizes the clinical and laboratory characteristics of the enrolled patients. Thromboembolic complications are common in SARS-CoV-2 infections and platelet counts and inflammation marker CRP were significantly elevated in COVID-19 patients. Furthermore, HDL cholesterol was decreased while apolipoprotein A-I was found elevated suggesting complex effects of SARS-CoV-2 on lipid metabolism.

### Stratification of COVID-19 patients using metabolomics and lipidomics

**Fig. 1:**
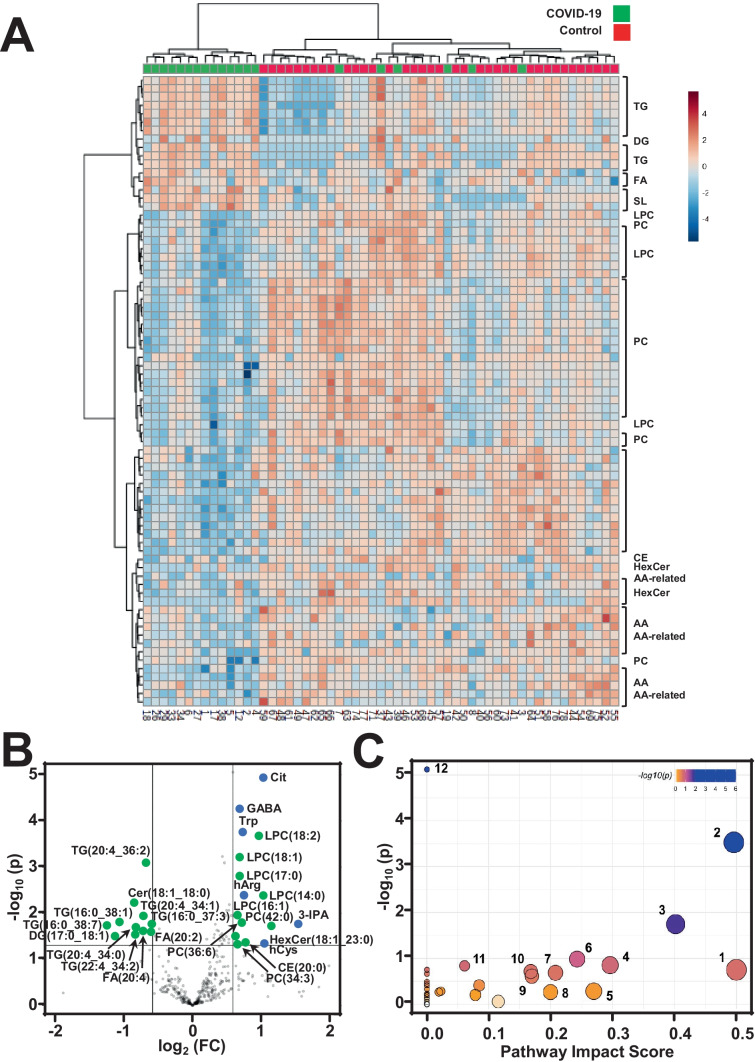
Metabolomics in COVID-19 patient plasma **A**. Heat map analysis of plasma metabolome analyzed in 20 COVID-19 and 37 control patients. Row displays metabolites and column represents the patient samples. Significantly decreased metabolites were displayed in blue, while metabolites significantly increased were displayed in red. The brightness of each color corresponded to the magnitude of the difference when compared with average value. Hierarchical cluster analysis shows the molecular species that are significantly different between the two groups including triglycerides (TG), diacylglycerols (DG), fatty acids (FA), sphingolipids (SL), lysophosphatidylcholines (LPC), phosphatidylcholines (PC), cholesteryl esters (CE), hexosylceramides (HexCer), and amino acids (AA). **B**. Volcano plot that gives the statistical significance (*p*-value) versus magnitude of change (log2(fold change)) of most different amino acid (blue dots) or lipid (green) metabolites in COVID-19 patients, as compared to controls. Abbreviations of metabolites are given in Suppl. Table 5. **C**. Metabolic pathway analysis plot demonstrates pathways that were most strongly disturbed in COVID-19 patients. Dots present the most relevant pathways. The pathway impact is presented by size and P-value by color. Dots are 1—phenylalanine, tyrosine and tryptophan biosynthesis; 2—sphingolipid metabolism; 3—glycine, serine and threonine metabolism; 4—arginine biosynthesis; 5—arachidonic acid metabolism; 6 – arginine and proline metabolism; 7—cysteine and methionine metabolism; 8—histidine metabolism; 9—glycerophospholipid metabolism; 10—glyoxylate and dicarboxylate metabolism; 11—alanine, aspartate and glutamate metabolism; 12—aminoacyl-tRNA biosynthesis

A hierarchical cluster analysis of identified metabolites revealed that COVID-19 and control patients clearly differed in their metabolic signatures, indicating that the observed metabolic alteration is indeed specific to COVID-19 patients. Statistical analysis confirmed 72 metabolites that were significantly different between the two groups (Suppl. Table 1). Distinct metabolites allowed for discrimination of COVID-19 and control plasma (Fig. 1A) and this was further highlighted by the scaled principal component analysis (PCA), which revealed metabolic phenotypes of sera from COVID-19-positive patients differing substantially from controls (Suppl. Fig. 1) Triglycerides, ceramides, sphingolipids and fatty acids were significantly elevated in COVID-19 patients whereas amino acid and glycerophospholipid plasma levels were reduced compared to control samples. Volcano plots highlighted the most distinct metabolites in COVID-19 plasma samples compared to controls (Fig. 1B). To identify disturbed pathways in COVID-19 patients, we performed a metabolic pathway analysis that revealed metabolic disturbances of amino acids tryptophan, tyrosine, arginine, and glutamate as well as sphingolipids and arachidonic acid as key metabolic pathways (Fig. 1C).

### Dysregulated amino acid metabolism in COVID-19

**Fig. 2:**
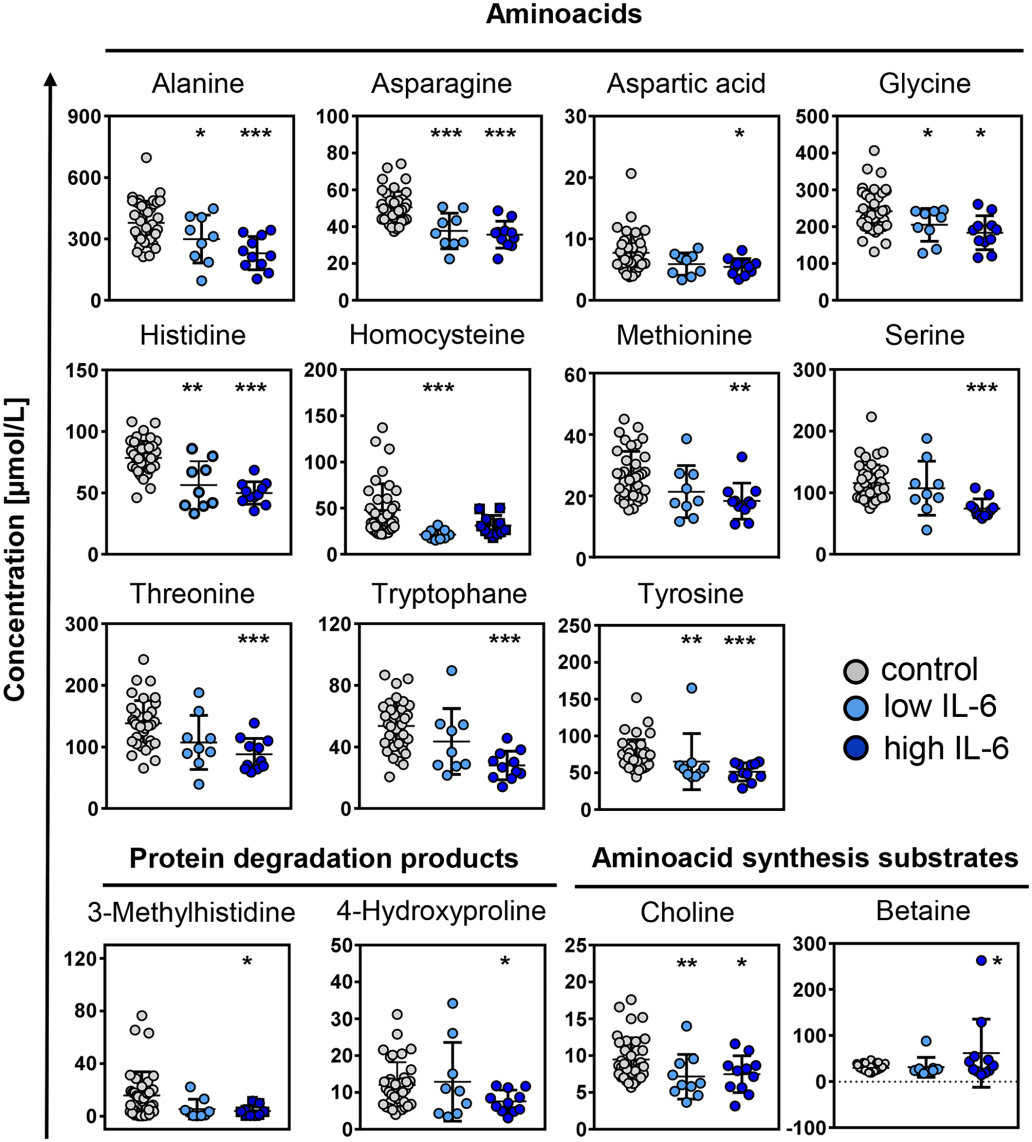
Amino acid levels in plasma of COVID-19 and control patientsPlasma levels of amino acids, proteolysis markers and amino acid synthesis substrates were determined by UPLC-MS/MS-based metabolomics in COVID-19 patients with low (< 15 pg/mL), and high (> 15 pg/mL) IL-6 levels or in control subjects. Data are expressed in µmol/L. Asterisks indicate levels of significance *p < 0.05; **p < 0.01; ***p < 0.001 *vs.* control patients

**Fig. 3:**
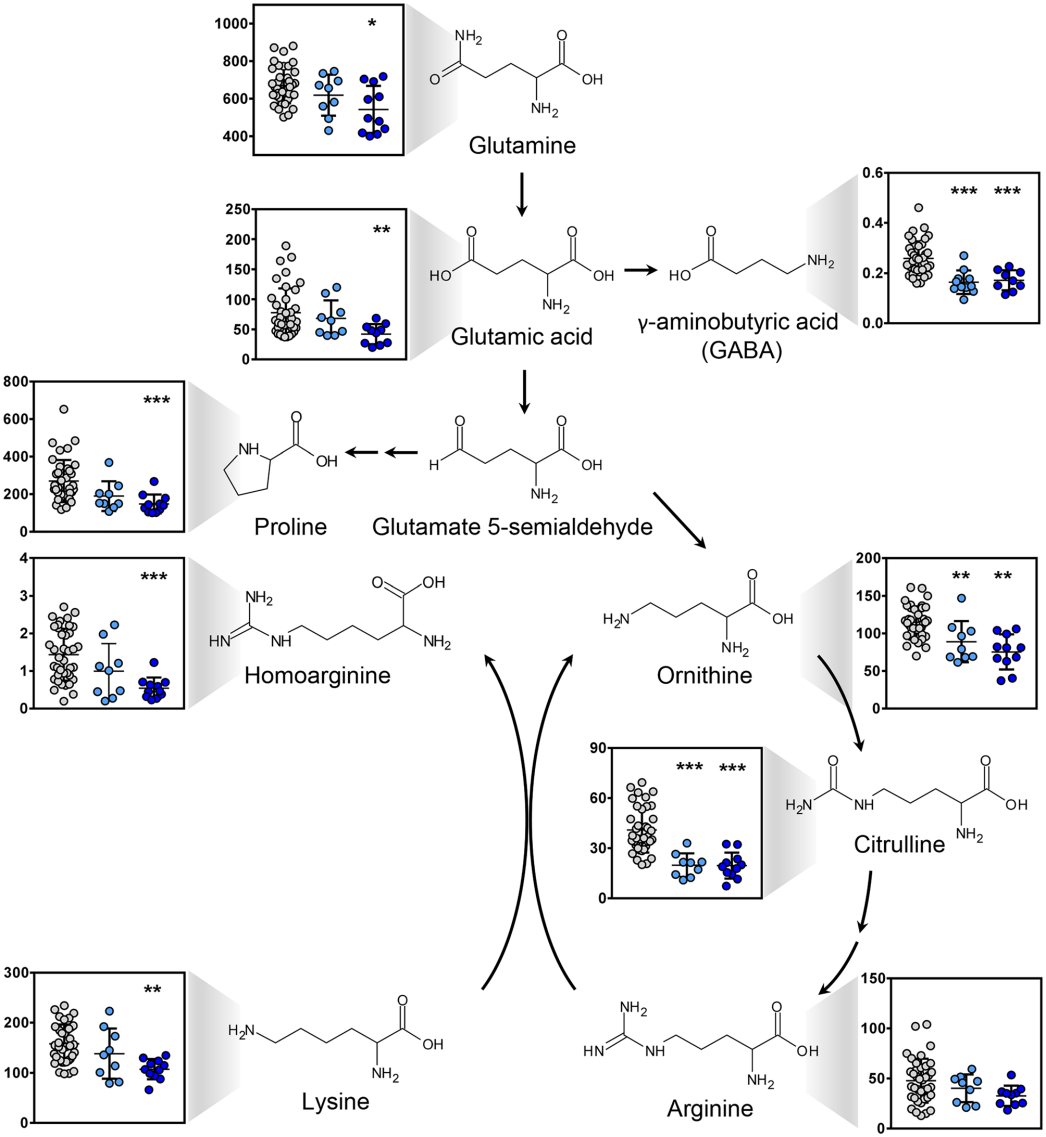
Plasma levels of glutamate and metabolites in COVID-19 patients. Plasma levels of amino acids, proteolysis markers and amino acid synthesis substrates were analyzed in COVID-19 patients with low (< 15 pg/mL, light blue dots) and high (> 15 pg/mL, dark blue dots) IL-6 levels and control patients (grey dots) by targeted metabolomics. Data are expressed in µmol/L. Glutamine and its metabolites were identified as the pathway most downregulated in SARS-CoV-2 infection. *p < 0.05; **p < 0.01; ***p < 0.001 compared to control group

High plasma IL-6 is associated with poor outcome in COVID-19 patients [17]. We sub-classified our COVID-19 patients according to their IL-6 levels assuming 15 ng/mL as a cut-off value reflecting the 95th percentile of IL-6 distribution in the control group. IL-6 was < 15 ng/L in 9 and > 15 ng/L in 11 patients, respectively. Decreases in plasma amino acid levels and IL-6 levels inversely correlated with each other and were more pronounced in the high IL-6 patient subgroup (Figs. 2 and 3). Consistently, IL-6 signal negatively correlated with plasma levels of most amino acids (Suppl. Table 2). Plasma levels of amino acids 3-methylhistidine and 4-hydroxyproline (Fig. 2), originating from actin/myosin and collagen metabolism [18], respectively, were also largely reduced in COVID-19 patients with high IL-6 levels.

### Alteration in tryptophan metabolism and indoleamine 2,3-dioxygenase activity

**Fig. 4:**
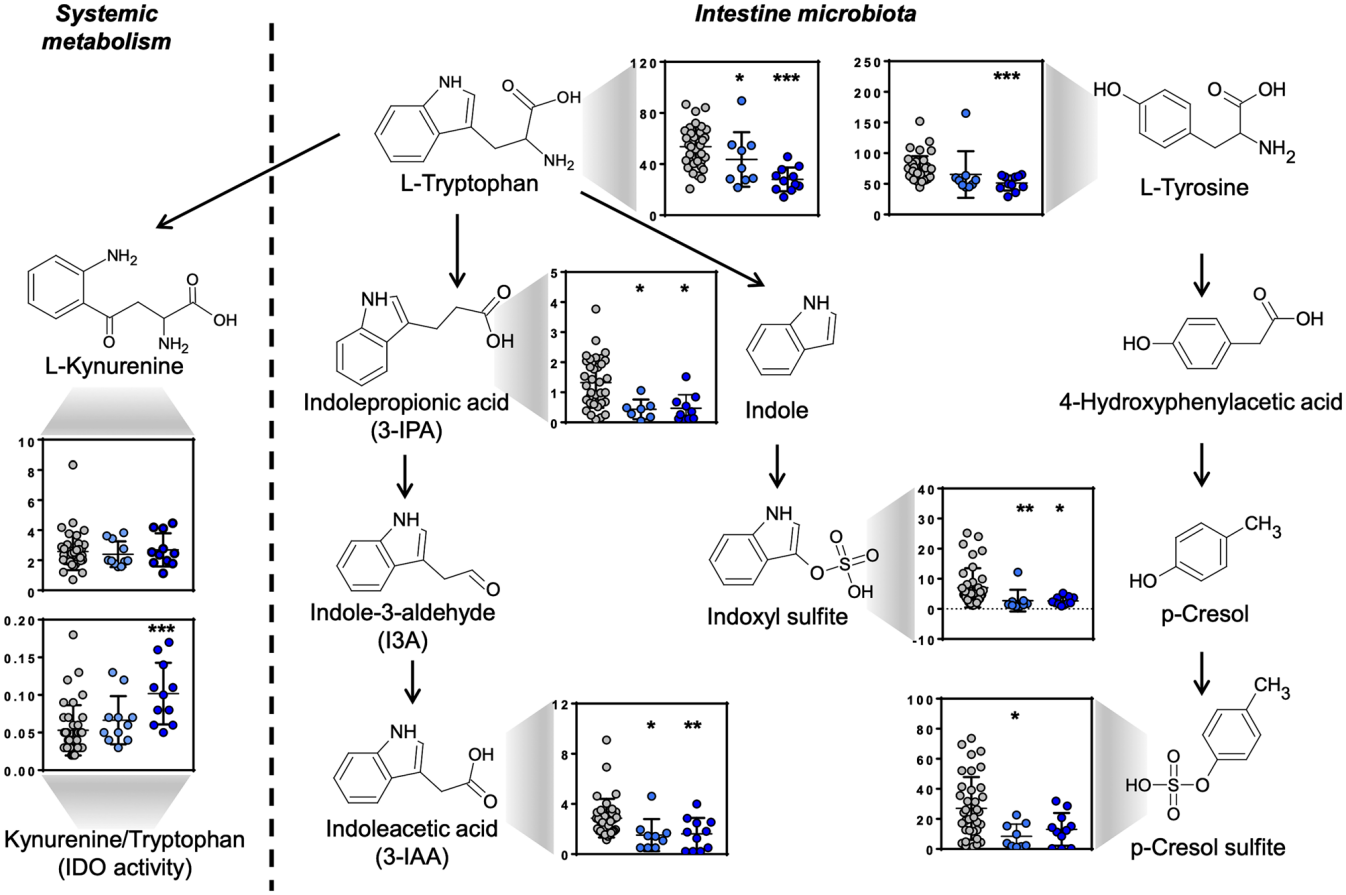
Plasma levels of systemic and gut microbiome-derived tryptophan and tyrosine metabolites in COVID-19 patients. Targeted metabolomics revealed reduced tryptophan and tyrosine pathway components in COVID-19 patients with low (< 15 pg/mL, light blue dots) and high (> 15 pg/mL, dark blue dots) IL-6 levels compared to control patients (grey dots). Data are expressed in µmol/L. Asterisks indicate levels of significance *vs.* controls *p < 0.05; **p < 0.01; ***p < 0.001

Tryptophan pathway was among the top pathways that was impacted by SARS-CoV-2 infection. The levels of tryptophan were significantly decreased in COVID-19 patients (Fig. 2) and were inversely correlated with IL-6 levels (Suppl. Table 2). The essential amino acid tryptophan catabolism is tightly controlled by the rate-limiting enzyme indoleamine 2,3-dioxygenase (IDO). IDO contributes to immune-metabolic regulation by depleting tryptophan or producing kynurenine, both contributing to an increased susceptibility to infection [19]. The kynurenine/tryptophan ratio can be used to estimate the activity of IDO. Although the plasma levels kynurenine was not significantly different between COVID-19-positive and COVID-19-negative patients, calculated IDO was significantly higher in COVID-19 patients (Fig. 4).

In addition, plasma levels of tryptophan degradation products by *Clostridia* (IPA), *Lactobacilli* (3-IAA) and tryptophanase-expressing bacteria (indoxyl sulfite) were diminished compared to controls (Fig. 4). Furthermore, plasma levels of p-cresol sulfate, which is a product of bacterial fermentation of proteins in the large intestine, were reduced in samples from SARS-CoV-2-infected patients. In contrast to tryptophan, most bacterial conversion products did not correlate with IL-6 levels;however, concentrations of these amino acid byproducts closely correlated with each other (Suppl. Table 2).

### Impairment of glutamate metabolism and arginine biosynthesis leads to reduction in plasma GABA in SARS-CoV-2-infected patients

Glutamine metabolism and arginine biosynthesis were also strongly modulated in COVID-19 patients and levels of GABA, citrulline and ornithine were reduced significantly compared to controls (Fig. 1B). However, there was not a significant difference in the level of circulating arginine between the two groups. In inflammatory disease, the rate of glutamine consumption by an array of immune cells is similar or even greater than glucose [20]. Furthermore, glutamine serves as a building block for various other amino acids [21] that were found reduced in SARS-CoV-2-infected patients (Fig. 3). The transmitter GABA is the major glutamine degradation product and was largely reduced in COVID-19 patient plasma. Similarly, citrulline that is intertwined with the glutamine metabolism was lower in samples from COVID-19 patients. Receiver operating characteristic (ROC) curves demonstrated that GABA and citrulline were the best predictive biomarkers in COVID-19-positive patient. GABA plasma levels < 0.214 μmol/L had the area under the curve (AUC) equal to 0.93 in distinguishing between SARS-CoV-2 infected and control patients and predictive power of GABA significantly exceeded that of IL-6 (Suppl. Fig. 2). Citrulline plasma levels < 33 µmol/L had the area under the curves equal to 0.92 and the predictive power borderline significantly better than IL-6 (Suppl. Fig. 2). Sensitivities and specificities at the cut-off values for 20 metabolites with highest AUCs are shown in Suppl. Table 4.

**Fig. 5:**
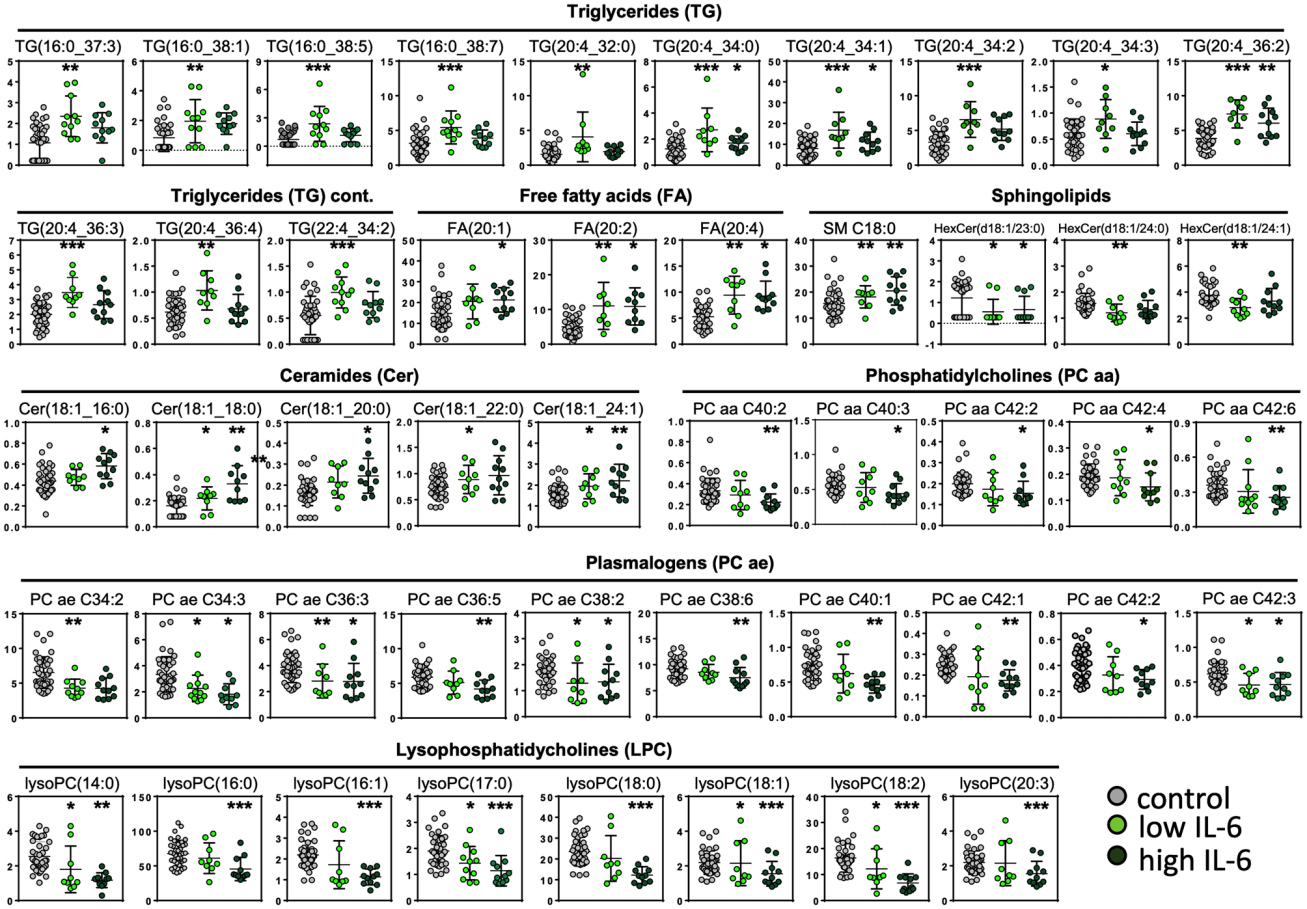
Deranged lipid metabolism in plasma of COVID-19 patients. Plasma levels of glycerophospholipids (phosphatidylcholines, lysophopsha-tidylcholines, plasmalogens), sphingolipids (sphingomyelins, ceramides, hexosylceramides), fatty acids and triglycerides were analyzed in COVID-19 patient plasma samples. Concentrations in COVID-19 with low (< 15 pg/mL) or high (> 15 pg/mL) IL-6 levels, and SARS-CoV-2-negative control patients. Data are expressed in µmol/L. Asterisks indicate significance compared to controls, *p < 0.05; **p < 0.01; ***p < 0.001

### IL-6 levels are inversely correlated with glycerophospholipid but not with sphingolipid metabolism in COVID-19 patients

For comprehensive insight into metabolic alterations induced by SARS-CoV-2, we analyzed impact of COVID-19-driven systemic inflammation for the lipid metabolism. Levels of glycerophospholipids including phosphatidylcholines, lysophosphatidylcholines and plasmalogens were significantly reduced in COVID-19 patients with high IL-6 levels (Fig. 5 and Suppl. Table 3). Similarly, the hexosylceramides HexCer(d18:1/24:0), HexCer(d18:1/24:1) and HexCer(d18:1/23:0) were lower in COVID-19 patients (data not shown). In contrast, plasma levels of an array of distinct lipid classes including triglycerides, fatty acids that are associated with the COVID-19 risk factor diabetes mellitus type 2 [22] as well as sphingomyelins and ceramides were increased in COVID-19 patient plasma (Fig. 5). Highest triglyceride levels were associated with low IL-6 concentrations (< 15 ng/L).

## Discussion

There are clear metabolic signatures in European patients with SARS-CoV-2 infections that require hospitalization. While several circulating amino acids and related metabolites were reduced, the tryptophan metabolism and arginine biosynthesis were among the top pathways impacted by SARS-CoV-2 infection (Fig. 1). Elevated IL-6 concentrations were associated with reduced tryptophan levels (Fig. 2), suggesting a regulation of inflammatory reactions to SARS-CoV-2 infection. In agreement with these observations, kynurenine has been previously associated with COVID-19 infections [23]. The essential amino acid tryptophan catabolism is tightly controlled by IDO. Tryptophan degradation has been described as an innate immune mechanism of host defence against infection [24]. IDO has immune-metabolic functions by regulating tryptophan or kynurenine, with implications for infections [19]. In addition, IDO-induction interfere with inflammation [25]. The kynurenine/tryptophan ratio can be used to estimate the activity of IDO. We observed that the plasma levels of kynurenine were not significantly different between COVID-19-positive and COVID-19-negative patients; however, calculated IDO was significantly higher in COVID-19 patients. [25, 26]. This suggests targeting IDO via IDO inhibitors [27] or tryptophan via nutritional supplementation may present a beneficial outcome for COVID-19 patients. To the best of our knowledge, our data show for the first time that SARS-CoV-2 infection interferes with the amino acid conversion by the gut microbiota (Fig. 4) in humans. In particular, the products of tryptophan and tyrosine metabolism including 3-IAA, IPA, indoxyl sulfate and p-cresol sulfate were substantially reduced in COVID-19 patients. Products of macrobiotic amino acid conversion were independent of the host inflammatory status indicating that SARS-CoV-2 pathology in the gut is at least partially uncoupled from the systemic inflammatory reaction. Clearly, the gut is a target organ for SARS-CoV-2 infection [28] and diarrhea is common in patients with COVID-19 [29, 30]. Consistently, the gut microbiome regulates COVID-19 disease [31] and increased levels of *Lactobacillus sp*. and *Ruminococcus gnavus* were associated with better and worse outcome, respectively. Metabolic activity of *Lactobacillus* sp. accounts for the conversion of tryptophan to IPA in the gut [32] and lower tryptophan levels are a key finding in our cohort of COVID-19 patients.

Catabolism of glutamate and arginine biosynthesis were largely defective in COVID-19 patients. Glutamate is a building block for synthesis of an array amino acids, and indeed levels of several amino acids derived from glutamate such as serine, aspartate, alanine, proline and tyrosine were reduced in SARS-CoV-2-infected patients (Fig. 2). Consistent with lower glutamate levels, its major metabolite GABA was also reduced in COVID-19 patient plasma. ROC curves for plasma levels of GABA below 0.214 µmol/L allowed for discrimination of COVID-19 patients with good sensitivity and specificity (AUC = 93%) significantly exceeding the predictive power of IL-6 (AUC = 78%, Suppl. Fig. 2). In addition to its neurotransmitter functions, GABA modulates immune reactions [33]. GABA protects beta-cells in pancreatic islets and plays an important role in diabetes [34]. Therefore, we checked whether the difference in GABA levels between COVID-19 patients and control subjects was still significant when patients with this comorbidity were excluded from statistical analysis. Indeed, the difference remained distinctive (p < 0.001). GABA receptors are expressed in various immune cells, including lymphocytes and dendritic cells, and regulate secretion of both pro- and anti-inflammatory cytokines from these cells [35]. In addition, lung epithelial cells express GABA receptors [36, 37] and GABA has the capacity for interference with inflammation and improves clearance of alveolar fluid in acute lung injury [38], suggesting a role of low GABA signaling in COVID-19-associated lung injury. Supporting a critical role of GABA in COVID-19-associated pneumonia, GABA administration attenuates severity of disease and reduced rate of death in an experimental coronavirus mouse hepatitis virus (MHV)-1 pneumonia model [39]. GABA was administered orally to infected mice and the agent was safe and efficiently absorbed by oral administration in humans [40], indicating a potential prophylactic or therapeutic application of the amino acid in COVID-19 patients [41]. Moreover, GABA stabilizes endothelial metabolism by regulating ATP synthesis as well as fatty acid and pyruvate oxidation [42]. Thus, the substantial reduction of GABA concentrations in plasma hospitalized COVID-19 patients documented in the present study for the first time is in line with endothelial dysfunction leading to thrombo-inflammation [43], a major disease burden in SARS-CoV-2 infections. GABA allows for stratification of COVID-19 patients; however, analysis of its clinical use requires further studies. Additionally, analysis of GABA in other viral respiratory disease, e.g., seasonal influenza will shed light on common pathways underlying viral pneumonia or identify pathogen-specific pathologies.

In line with our findings, low levels of circulating amino acids constitute a hallmark of coronavirus infections [16, 44], including SARS-CoV-1 and MERS-CoV, but also Ebola virus [45, 46] suggesting a generalized host response pattern to viral infection. The underlying mechanisms appears multi-factorial including excess consumption, phagocytosis by immune cells and disturbed liver metabolisms. Similar effects including alterations in glutamine and glutamate metabolism, depletion of tryptophan and increased cellular IDO activity were observed in in A549 and nasal epithelial cells upon influenza virus infection, which is like SARS-CoV-2 an enveloped, single-stranded RNA virus triggering a cytokine storm [47, 48]. Supporting the notion that SARS-CoV-2 directly affects hepatocytes [49], liver-derived PCs, plasmalogens and LPCs were lower in COVID-19 plasma. Liver biomarkers LDL, HDL and apo A-I were reduced in MERS-CoV, Ebola and Dengue infections [50, 51]. Furthermore, increased sphingolipid concentrations observed in our COVID-19 patients [52] (Fig. 5) are in line with hepatic sphingolipid synthesis and secretion into plasma in form of exosomes [53]. Sphingolipids are membrane-bound potent bioactive lipids involved in the pathogenesis of various respiratory bacterial infections and regulate host–pathogen interactions. Ceramides and sphingosine-1-phosphate (S1P) are key lipids generated within this pathway. Several studies have demonstrated that sphingolipids are critical mediators positively or negatively affecting inflammatory processes in several respiratory diseases including asthma, cystic fibrosis (CF), chronic obstructive pulmonary disease (COPD). There is increasing evidence that S1P is also pivotal in the regulation of immune cell activation, trafficking and inflammation, which makes it an excellent candidate for therapeutic targeting [54]. Taken together, our findings indicate that the liver is an important target organ in SARS-CoV-2 infection. Of note, classical liver biomarkers such as ALT and bilirubin did not differ or were found even lower in the COVID-19 group as compared to controls, highlighting the need of novel biomarkers in SARS-CoV-2 infection.

Collectively, our results show that SARS-CoV-2 infection is associated with a plethora of metabolic alterations in patient plasma. As profound metabolic disturbances in COVID-19 patients are associated with only minor changes in routine laboratory parameters, we hypothesize that some of them may be used with caution as useful indicators of the disease severity and outcome. Furthermore, these alterations, if reversed, might be suitable to induce a positive outcome of SARS-CoV-2 infections. Clearly further validation is needed prior to testing GABA-based therapy in clinical trials.

We would like to emphasize some limitations of the current study. First, the cohort size with only 20 COVID patient is comparably small. Second, patients in the COVID-19 suffered disproportionally from diabetes mellitus, which is known to exert profound effect on intermediary metabolism, and which could spuriously contribute to metabolic alterations in course of SARS-CoV-2 infection. However, no overt clustering of COVID-19 patients and control subjects with diabetes was noted in the PCA analysis arguing against the notion that this condition acts as a major driver of metabolic distortion observed in COVID-19. In addition, the concentration of GABA and Cit—two predictive markers revealed in the present study—was similar in the COVID-19 patients regardless of their diabetic status (Suppl. Fig. 3). In this context, it is worth noticing that SARS-CoV-2 infection and diabetes exerts opposite effects on several metabolites, which were subject to alterations in the COVID-19 group. For instance, elevated plasma concentrations of several amino acids including tryptophan (and its metabolites), tyrosine, phenylalanine, glutamate and alanine were found in diabetic subjects. We hypothesize that the up-regulating effect of diabetes on these amino acid levels was fully outweighed by the down-regulating effect of SARS-CoV-2 infection in population examined in the present study. Third, we cannot entirely exclude that some changes of the microbiome-derived metabolites, which were seen in the COVID-19 patients, may be attributed to the specific adaptation to hospital diet in this group, even though control patients in our study were treated in the same hospital and received the same meals as their SARS-CoV-2-infected counterparts. However, Zou et al. [28] recently reported that an altered intestinal microbiome in COVID-19 patients was independent from the hospital meals. Finally, although the analytical approach used for this study had a good coverage for amino acid metabolism, we believe the assessment of lipid metabolism could improve significantly by using more specific lipidomic approach allowing identification of additional predictive markers.

Despite these limitations, we are confident that investigating metabolism in COVID-19 patients using a comprehensive metabolomics approach can provide valuable and novel insights and facilitate the tailored strategy for treating patients, paving the path to precision medicine. The study design was done in a manner to remove any potential bias related to sample collection, dietary intake, age, etc. A streamlined sample collection and subsequent metabolomics workflow could deliver data within a short timeframe. To the best of our knowledge, this is the first report of reduced GABA and related metabolites in hospitalized patients with SARS-CoV-2 infection. Further clinical studies are required to validate the metabolic signature, potential predictive markers as a basis to develop new therapeutic interventions.

**Table 1:** Demographic and clinical characteristics of COVID-19 and control groups. Data are shown as arithmetic means (± SD) or geometric means with 95% CI for the mean back-transformed after logarithmic transformation. For discrete or continuous variables significance was determined with χ^2^ or Student’s and Welch’s t-tests

			**COVID-19**	**Control**	***p*** **value**
**Demographic characteristics**					
Age (years)			59.2 ± 18.2	63.3 ± 16.3	0.34
Gender					
	Male (n)		10	23	0.64
	Female (n)		10	14	0.57
**Comorbidities,** ***n*** **(%)**					
Diabetes mellitus			8 (40)	2 (5)	** < 0.001**
Hypertension			7 (35)	10 (27)	0.64
Asthma bronchiale			3 (15)	4 (11)	0.68
Cancer			6 (30)	9 (24)	0.72
Renal diseases			3 (15)	4 (11)	0.64
Coronary heart disease			2 (10)	9 (24)	0.27
Other heart diseases			3 (15)	10 (27)	0.41
Thyroid dysfunction			3 (15)	6(16)	0.91
Cholecystolithiasis			1 (5)	3(8)	0.68
**Symptoms,** ***n*** **(%)**					
Dyspnea			6(30)	4 (11)	0.14
Fever at metabolomic screening			1 (5)	0 (0)	0.18
Fever during hospitalization			11 (55)	1 (3)	**0.004**
Diarrhea			3 (15)	0 (0)	**0.03**
Dry cough			8 (40)	1 (3)	**0.003**
Headache			2 (10)	0 (0)	0.06
Fatigue			5 (25)	1 (3)	**0.03**
None of above symptoms			4 (20)	28 (76)	**0.02**
**Diagnostic imaging,** ***n*** **(%)**					
Pulmonary infiltrates			16 (80)	2 (5)	** < 0.001**
**Laboratory parameters**					
Hb (g/L)			12.3 ± 6.0	11.8 ± 2.5	0.78
Red blood cells (T/L)			4.06 ± 0.73	3.56 ± 0.71	**0.02**
White blood cells (G/L)			6.8 ± 3.3	6.5 ± 3.2	0.47
Platelets (G/L)			285 ± 99	222 ± 102	**0.03**
Sodium (mmol/L)			140 ± 4	141 ± 3	0.43
Potassium (mmol/L)			3.8 ± 0.5	3.9 ± 0.3	0.25
AST (U/l)			21.7 < 29.1 < 39.1	16.8 < 20.3 < 24.5	**0.04**
ALT (U/l)			27.3 ± 16.1	23.0 ± 11.8	0.08
CRP (mg/l)			17.7 < 34.4 < 67.7	4.0 < 4.2 < 4.4	** < 0.001**
IL-6 (ng/L)			4.6 < 5.7 < 7.6	11.3 < 21.3 < 39.8	** < 0.001**
Bilirubin (mg/dL)			0.29 ± 0.14	0.48 ± 0.29	**0.018**
Creatinine (mg/dl)			0.67 < 1.06 < 1.67	0.85 < 1.00 < 1.18	0.82
Blood urea nitrogen (mg/dL)			7.7 < 12.0 < 18.8	14.3 < 18.2 < 23.3	0.65
Total cholesterol (mg/dL)			155.9 ± 3.3	162.6 ± 33.7	0.52
Triglycerides (mg/mL)			119 < 144 < 174	98 < 113 < 1443	0.07
HDL-cholesterol (mg/dL)			31.2 ± 10.9	42.6 ± 14.3	**0.003**
Apolipoprotein A-I (mg/dL)			106.3 ± 23.1	80.2 ± 20.9	** < 0.001**

## Supplementary information

Below is the link to the electronic supplementary material.

Supplementary file1 (DOCX 748 KB)

## Data Availability

The datasets generated during and/or analyzed during the current study are available from the corresponding author on reasonable request. The mass spectrometry metabolomics data have been deposited to MetaboLights as MTBLS3515 (https://www.ebi.ac.uk/metabolights/MTBLS3515).
