# Supplementary material for: Disturbed lipid and amino acid metabolisms in COVID-19 patients
Source: J Mol Med (Berl). 2022 Jan 22;100(4):555–68. doi: 10.1007/s00109-022-02177-4 (PMC8783191; doi:10.1007/s00109-022-02177-4)

**Supplemental Data**

"Disturbed Lipid and Amino Acid Metabolisms in COVID-19 patients"

*Journal of Molecular Medicine*

Mojgan Masoodi; Manuela Peschka; Stefan Schmiedel; Munif Haddad; Maike Frye; Coen Maas; Ansgar Lohse; Samuel Huber; Paulus Kirchhof; Jerzy-Roch Nofer; Thomas Renné

**Supplemental Table 1**. Metabolites differing between patients with SARS-CoV2 infection and controls.

| **Metabolite** | **FDR adjusted p-value** |
| --- | --- |
| Cit | 2.60E-05 |
| GABA | 7.64E-05 |
| His | 2.39E-04 |
| lysoPC a C18:2 | 2.54E-04 |
| Trp | 2.54E-04 |
| lysoPC a C18:1 | 8.81E-04 |
| Asn | 9.86E-04 |
| Orn | 1.16E-03 |
| Tyr | 1.87E-03 |
| lysoPC a C14:0 | 2.75E-03 |
| lysoPC a C17:0 | 2.75E-03 |
| lysoPC a C16:1 | 2.75E-03 |
| 5-AVA | 3.64E-03 |
| PC ae C34:2 | 3.64E-03 |
| C0 | 4.09E-03 |
| HArg | 4.10E-03 |
| lysoPC a C18:0 | 5.14E-03 |
| HexCer(d18:1/24:1) | 5.14E-03 |
| Cer(d18:1/18:0) | 5.14E-03 |
| Met | 6.76E-03 |
| PC ae C36:3 | 7.81E-03 |
| PC ae C40:1 | 9.22E-03 |
| TG(20:4_36:2) | 9.22E-03 |
| PC ae C34:3 | 9.86E-03 |
| Ala | 9.86E-03 |
| Pro | 1.00E-02 |
| lysoPC a C16:0 | 1.00E-02 |
| TG(16:0_37:3) | 1.00E-02 |
| 3-IPA | 1.01E-02 |
| PC aa C42:2 | 1.22E-02 |
| PC aa C42:6 | 1.37E-02 |
| TG(16:0_38:1) | 1.50E-02 |
| Choline | 1.63E-02 |
| Trigonelline | 1.64E-02 |
| HexCer(d18:1/23:0) | 1.64E-02 |
| CE(20:0) | 1.64E-02 |
| HCys | 1.64E-02 |
| PC aa C32:2 | 1.64E-02 |
| PC ae C42:3 | 1.64E-02 |
| Thr | 1.64E-02 |
| HexCer(d18:1/24:0) | 1.64E-02 |
| TG(20:4_34:0) | 1.64E-02 |
| TG(20:4_34:1) | 1.64E-02 |
| Ser | 1.68E-02 |
| ArA | 1.74E-02 |
| PC aa C40:2 | 1.81E-02 |
| Lys | 1.81E-02 |
| TG(22:4_34:2) | 2.01E-02 |
| AconAcid | 2.43E-02 |
| PC ae C42:2 | 2.46E-02 |
| PC ae C38:6 | 2.46E-02 |
| PC ae C36:5 | 2.55E-02 |
| PC ae C42:1 | 2.86E-02 |
| lysoPC a C20:3 | 2.89E-02 |
| FA(20:2) | 3.12E-02 |
| TG(16:0_38:7) | 3.12E-02 |
| 3-IAA | 3.25E-02 |
| PC ae C38:2 | 3.50E-02 |
| TrpBetaine | 3.91E-02 |
| DG(17:0_18:1) | 3.91E-02 |
| PC aa C36:6 | 4.05E-02 |
| PC aa C34:4 | 4.05E-02 |
| PC aa C38:0 | 4.05E-02 |
| PC ae C30:0 | 4.05E-02 |
| PC ae C42:0 | 4.59E-02 |
| PC ae C38:0 | 4.65E-02 |
| Gly | 4.65E-02 |
| Cer(d18:1/24:1) | 4.65E-02 |
| TG(20:4_36:3) | 4.65E-02 |
| Hex2Cer(d18:1/20:0) | 4.65E-02 |
| TG(20:4_34:2) | 4.69E-02 |
| TG(16:0_38:5) | 4.75E-02 |

**Supplemental Table 2**. Correlations between amino acids and amino acid microbiotic products in COVID-19 patients

|  | **3-IAA** | **3-IPA** | **Ala** | **Arg** | **Asn** | **Asp** | **Cit** | **Gln** | **Glu** | **Gly** | **His** | **Ind**  **SO4** | **Lys** | **Met** | **Orn** | **pCre**  **SO4** | **Pro** | **Ser** | **Thr** | **Trp** | **Tyr** |
| --- | --- | --- | --- | --- | --- | --- | --- | --- | --- | --- | --- | --- | --- | --- | --- | --- | --- | --- | --- | --- | --- |
| **3IPA** | 0.65*** |  |  |  |  |  |  |  |  |  |  |  |  |  |  |  |  |  |  |  |  |
| **Ala** | 0.16 | 0.42* |  |  |  |  |  |  |  |  |  |  |  |  |  |  |  |  |  |  |  |
| **Arg** | 0.27 | 0.13 | 0.47** |  |  |  |  |  |  |  |  |  |  |  |  |  |  |  |  |  |  |
| **Asn** | 0.28 | 0.31 | 0.55*** | 0.79*** |  |  |  |  |  |  |  |  |  |  |  |  |  |  |  |  |  |
| **Asp** | 0.07 | 0.14 | 0.52*** | 0.06 | 0.13 |  |  |  |  |  |  |  |  |  |  |  |  |  |  |  |  |
| **Cit** | 0.16 | 0.35 | 0.59*** | 0.54*** | 0.67*** | 0.29 |  |  |  |  |  |  |  |  |  |  |  |  |  |  |  |
| **Gln** | 0.3 | 0.15 | 0.68*** | 0.47** | 0.65*** | 0.39* | 0.42** |  |  |  |  |  |  |  |  |  |  |  |  |  |  |
| **Glu** | -0.05 | 0.09 | 0.35* | 0.22 | 0.14 | 0.73*** | 0 | 0.193 |  |  |  |  |  |  |  |  |  |  |  |  |  |
| **Gly** | 0.1 | 0.05 | 0.48** | 0.57*** | 0.59*** | 0.11 | 0.41** | 0.70* | 0.05 |  |  |  |  |  |  |  |  |  |  |  |  |
| **His** | 0.43* | 0.58** | 0.62*** | 0.52*** | 0.76*** | 0.25 | 0.64*** | 0.53*** | 0.05 | 0.29 |  |  |  |  |  |  |  |  |  |  |  |
| **Ind**  **SO4** | 0.42* | 0.68*** | 0.38* | 0.23 | 0.35* | 0.15 | 0.57*** | 0.09 | 0.16 | 0.03 | 0.55*** |  |  |  |  |  |  |  |  |  |  |
| **Lys** | 0.21 | 0.26 | 0.46** | 0.80*** | 0.80*** | 0.18 | 0.55*** | 0.43* | 0.03 | 0.52*** | 0.63*** | 0.18 |  |  |  |  |  |  |  |  |  |
| **Met** | 0.37* | 0.321 | 0.44** | 0.67*** | 0.85** | 0.14 | 0.63*** | 0.54*** | -0.08 | 0.39* | 0.81*** | 0.38* | 0.79*** |  |  |  |  |  |  |  |  |
| **Orn** | 0.13 | 0.22 | 0.34* | 0.40* | 0.50** | 0.17 | 0.75*** | 0.24 | 0.04 | 0.09 | 0.55*** | 0.41* | 0.38* | 0.54*** |  |  |  |  |  |  |  |
| **pCre**  **SO4** | 0.43* | 0.73*** | 0.32* | 0.25 | 0.36 | 0.26 | 0.50** | 0.12 | 0.07 | 0.04 | 0.61*** | 0.74* | 0.38* | 0.47** | 0.48** |  |  |  |  |  |  |
| **Pro** | 0.24 | 0.28 | 0.75*** | 0.60*** | 0.643 | 0.42 | 0.74*** | 0.72*** | 0.11 | 0.59*** | 0.60*** | 0.47** | 0.53*** | 0.60*** | 0.50*** | 0.3 |  |  |  |  |  |
| **Ser** | 0.39* | 0.32 | 0.62*** | 0.62*** | 0.746 | 0.19 | 0.49** | 0.72*** | 0.12 | 0.61*** | 0.65*** | 0.18 | 0.69*** | 0.75*** | 0.28 | 0.179 | 0.62*** |  |  |  |  |
| **Thr** | 0.04 | 0.15 | 0.52*** | 0.69*** | 0.87 | 0.18 | 0.52*** | 0.72*** | 0.04 | 0.68*** | 0.63*** | 0.13 | 0.75*** | 0.75*** | 0.34* | 0.194 | 0.65*** | 0.69*** |  |  |  |
| **Trp** | 0.11 | 0.45* | 0.56*** | 0.14 | 0.492 | 0.24 | 0.49** | 0.29 | 0.19 | 0.13 | 0.61*** | 0.33* | 0.36* | 0.49*** | 0.49** | 0.282 | 0.48** | 0.54*** | 0.38* |  |  |
| **Tyr** | 0.22 | 0.52** | 0.42** | 0.37* | 0.567 | 0.28 | 0.55*** | 0.22 | 0.11 | 0.16 | 0.64*** | 0.60** | 0.59*** | 0.65*** | 0.44** | 0.69* | 0.41** | 0.42** | 0.46** | 0.58*** |  |
| **IL-6** | 0.09 | 0.05 | -0.67*** | -0.26 | -0.41* | -0.48** | -0.43** | -0.76*** | -0.39* | -0.57*** | -0.29 | 0.07 | -0.43** | -0.35* | -0.29 | 0.01 | -0.57*** | -0.63*** | -0.61*** | -0.36* | -0.21 |

Significance was determined by the non-parametric Spearman correlation test. Asterisks indicate significance *p < 0.05; **p < 0.01; ***p < 0.001.

|  | **lysoPC**  **(14:0)** | **lysoPC**  **(16:0)** | **lysoPC**  **(16:1)** | **lysoPC**  **(17:0)** | **lysoPC**  **(C18:0)** | **lysoPC**  **(C18:1)** | **lysoPC**  **(18:2)** | **lysoPC**  **(20:3)** | **GABA** | **HArg** | **Cer**  **(18:1_16:0)** | **Cer**  **(18:1_18:0)** | **Cer**  **(18:1_20:0)** | **Cer**  **(18:1_22:0)** | **Cer**  **(18:1_24:1)** |
| --- | --- | --- | --- | --- | --- | --- | --- | --- | --- | --- | --- | --- | --- | --- | --- |
| **lysoPC(16:0)** | 0.64*** |  |  |  |  |  |  |  |  |  |  |  |  |  |  |
| **lysoPC(16:1)** | 0.75*** | 0.82`*** |  |  |  |  |  |  |  |  |  |  |  |  |  |
| **lysoPC(17:0)** | 0.83*** | 0.83*** | 0.77*** |  |  |  |  |  |  |  |  |  |  |  |  |
| **lysoPC(18:0)** | 0.71*** | 0.93*** | 0.81*** | 0.83*** |  |  |  |  |  |  |  |  |  |  |  |
| **lysoPC(C18:1)** | 0.75*** | 0.94*** | 0.84*** | 0.90*** | 0.90*** |  |  |  |  |  |  |  |  |  |  |
| **lysoPC(18:2)** | 0.76*** | 0.87*** | 0.78*** | 0.82*** | 0.86*** | 0.93*** |  |  |  |  |  |  |  |  |  |
| **lysoPC(20:3)** | 0.81*** | 0.80*** | 0.88*** | 0.88*** | 0.78*** | 0.85*** | 0.80*** |  |  |  |  |  |  |  |  |
| **GABA** | 0.29 | 0.55** | 0.50** | 0.41* | 0.61** | 0.51** | 0.42* | 0.44** |  |  |  |  |  |  |  |
| **HArg** | 0.49`` | 0.59*** | 0.49** | 0.6*** | 0.60*** | 0.63*** | 0.59*** | 0.52** | 0.44* |  |  |  |  |  |  |
| **Cer(18:1­_16:0)** | -0.14 | -0.19 | -0.13 | -0.01 | -0.16 | -0.20 | -0.27 | 0.06 | -0.25 | -0.17 |  |  |  |  |  |
| **Cer(18:1_18:0)** | -0.36* | -0.10 | -0.15 | -0.19 | 0.02 | -0.25 | -0.32 | -0.12 | 0.029 | -0.12 | 0.53** |  |  |  |  |
| **Cer(18:1_20:0)** | -0.18 | -0.09 | -0.06 | -0.14 | -0.20 | -0.29 | 0.05 | -0.15 | -0.06 | -0.26 | 0.58*** | 0.78*** |  |  |  |
| **Cer(18:1_22:0)** | 0.07 | -0.07 | 0.09 | -0.13 | -0.05 | -0.09 | 0.30 | -0.06 | -0.19 | -0.03 | 0.74** | 0.38* | 0.72*** |  |  |
| **Cer(18:1_24:1)** | -0.17 | -0.21 | -0.07 | -0.07 | -0.25 | -0.32 | 0.10 | -0.18 | -0.23 | -0.25 | 0.86*** | 0.50** | 0.72*** | 0.83*** |  |
| **IL6** | -0.39* | -0.69*** | -0.50** | -0.74*** | -0.66*** | -0.77*** | -0.43** | -0.68*** | -0.48** | -0.34* | 0.38 | 0.19 | 0.09 | 0.09 | 0.29 |

**Supplemental Table 3**. Correlations between metabolites with protective and deleterious effects

on endothelial function in COVID-19 patients

Significance was determined by the non-parametric Spearman correlation test. Asterisks indicate significance *p < 0.05; **p < 0.01; ***p < 0.001.

**Supplemental Table 4.** ROC analysis: sensitivity and specificity at the cut-off values for 20 metabolites with highest AUC values.

| **Metabolite** | **AUC** | **C.I.** | **Cutt-off** | **Sensitivity**  **(%)** | **Specificity**  **(%)** |
| --- | --- | --- | --- | --- | --- |
| IL-6 | 0.78 | 0.65 – 0.88 | 15.5 | 59 | 97 |
| **Metabolites** | | | | | |
| GABA | 0.93 | 0.79 – 0.96 | 0.21 | 73 | 95 |
| Cit | 0.92 | 0.82 – 0.94 | 33.0 | 100 | 69 |
| His | 0.88 | 0.77 – 0.95 | 68.7 | 90 | 79 |
| Tyr | 0.84 | 0.73 – 0.92 | 65.2 | 95 | 72 |
| lysoPC(18:2) | 0.81 | 0.69 – 0.90 | 9.05 | 70 | 94 |
| Trp | 0.81 | 0.70 – 0.90 | 36.7 | 70 | 87 |
| Orn | 0.81 | 0.68 – 0.89 | 82.3 | 60 | 95 |
| 5-AVA | 0.80 | 0.69 – 0.90 | 0.04 | 80 | 74 |
| C0 | 0.79 | 0.67 – 0.89 | 26.2 | 65 | 85 |
| Cer(18:1_18:0) | 0.78 | 0.66 – 0.88 | 0.19 | 85 | 67 |
| hArg | 0.77 | 0.64 – 0.86 | 0.71 | 65 | 85 |
| lysoPC(18:1) | 0.77 | 0.65 – 0.87 | 12.0 | 65 | 95 |
| PC ae C34:2 | 0.76 | 0.59 – 0.83 | 3.14 | 65 | 77 |
| lysoPC(14:0) | 0.75 | 0.62 – 0.86 | 1.32 | 63 | 97 |
| lysoPC(17:0) | 0.75 | 0.62 – 0.85 | 1.30 | 60 | 89 |
| Met | 0.75 | 0.62 – 0.85 | 18.4 | 60 | 90 |
| lysoPC(16:1) | 0.74 | 0.61 – 0.85 | 1.65 | 75 | 87 |
| lysoPC(18:0) | 0.74 | 0.61 – 0.84 | 17.9 | 70 | 85 |
| PC ae C36:3 | 0.72 | 0.59 – 0.83 | 3.14 | 65 | 77 |
| PC ae C40:1 | 0.72 | 0.59 – 0.83 | 0.62 | 75 | 69 |

Cut-off is expressed in µmol/L for all metabolites except IL-6 (pg/mL) and ratios.

**Supplemental Table 5.** List of abbreviations

| 3-IAA | indoleacetic acid |
| --- | --- |
| 3-IPA | indolepropionic acid |
| 5-AVA | 5-aminovaleric acid |
| ArA | arachidonic acid |
| AA | amino acid |
| AconAcid | aconitic acid |
| Ala | alanine |
| Asn | asparagine |
| C0 | carnitine |
| CE(20:0) | cholesteryl ester 20:0 |
| Cer(d18:1/18:0) | ceramide(d18:1/18:0) |
| Cer(d18:1/24:1) | ceramide(d18:1/24:1) |
| Cit | citrulline |
| DG(17:0_18:1) | diacylglyceride(17:0_18:1) |
| FA(20:2) | eicosadienoic acid |
| GABA | gamma-aminobutyric acid |
| Gly | glycine |
| HArg | homoarginine |
| HCys | homocysteine |
| Hex2Cer(d18:1/20:0) | dihexosylceramide(d18:1/20:0) |
| HexCer(d18:1/23:0) | hexosylceramide(d18:1/23:0) |
| HexCer(d18:1/24:0) | hexosylceramide(d18:1/24:0) |
| HexCer(d18:1/24:1) | hexosylceramide(d18:1/24:1) |
| His | histidine |
| Lys | lysine |
| lysoPC a C14:0 | lysophosphatidylcholine a C14:0 |
| lysoPC a C16:0 | lysophosphatidylcholine a C16:0 |
| lysoPC a C16:1 | lysophosphatidylcholine a C16:1 |
| lysoPC a C17:0 | lysophosphatidylcholine a C17:0 |
| lysoPC a C18:0 | lysophosphatidylcholine a C18:0 |
| lysoPC a C18:1 | lysophosphatidylcholine a C18:1 |
| lysoPC a C18:2 | lysophosphatidylcholine a C18:2 |
| lysoPC a C20:3 | lysophosphatidylcholine a C20:3 |
| Met | methionine |
| Orn | ornithine |
| PC aa C32:2 | phosphatidylcholine aa C32:2 |
| PC aa C34:4 | phosphatidylcholine aa C34:4 |
| PC aa C36:6 | phosphatidylcholine aa C36:6 |
| PC aa C38:0 | phosphatidylcholine aa C38:0 |
| PC aa C40:2 | phosphatidylcholine aa C40:2 |
| PC aa C42:2 | phosphatidylcholine aa C42:2 |
| PC aa C42:6 | phosphatidylcholine aa C42:6 |
| PC ae C30:0 | phosphatidylcholine ae C30:0 |
| PC ae C34:2 | phosphatidylcholine ae C34:2 |
| PC ae C34:3 | phosphatidylcholine ae C34:3 |
| PC ae C36:3 | phosphatidylcholine ae C36:3 |
| PC ae C36:5 | phosphatidylcholine ae C36:5 |
| PC ae C38:0 | phosphatidylcholine ae C38:0 |
| PC ae C38:2 | phosphatidylcholine ae C38:2 |
| PC ae C38:6 | phosphatidylcholine ae C38:6 |
| PC ae C40:1 | phosphatidylcholine ae C40:1 |
| PC ae C42:0 | phosphatidylcholine ae C42:0 |
| PC ae C42:1 | phosphatidylcholine ae C42:1 |
| PC ae C42:2 | phosphatidylcholine ae C42:2 |
| PC ae C42:3 | phosphatidylcholine ae C42:3 |
| Pro | proline |
| Ser | serine |
| TG(16:0_37:3) | triacylglyceride(16:0_37:3) |
| TG(16:0_38:1) | triacylglyceride(16:0_38:1) |
| TG(16:0_38:5) | triacylglyceride(16:0_38:5) |
| TG(16:0_38:7) | triacylglyceride(16:0_38:7) |
| TG(20:4_34:0) | triacylglyceride(20:4_34:0) |
| TG(20:4_34:1) | triacylglyceride(20:4_34:1) |
| TG(20:4_34:2) | triacylglyceride(20:4_34:2) |
| TG(20:4_36:2) | triacylglyceride(20:4_36:2) |
| TG(20:4_36:3) | triacylglyceride(20:4_36:3) |
| TG(22:4_34:2) | triacylglyceride(22:4_34:2) |
| Thr | threonine |
| Trp | tryptophan |
| TrpBetaine | tryptophan betaine |
| Tyr | tyrosine |
| COVID-19 | coronavirus disease 2019 |
| IL | interleukin |
| ROC | Receiver operating characteristic |
| SARS-CoV-2 | severe acute respiratory syndrome coronavirus 2 |
| CRP | C-reactive protein |
| ALT | alanine aminotransferase |
| AST | aspartate aminotransferase |
| TNF-α | tumour necrosis factor alpha |
| IP-10 | interferon-γ-induced protein 10 |
| MCP-1 | monocyte chemoattractant protein 1 |
| PCR | polymerase chain reaction |
| EDTA | ethylenediaminetetraacetic acid |
| ECLIA | electro-chemiluminescence immunoassay |
| apo A-I | apolipoprotein A-I |
| HDL | high-density lipoprotein |
| UPLC | ultra high pressure liquid chromatography |
| MS | mass spectrometry |
| IDO | indoleamine 2,3-dioxygenase |
| MHV | mouse hepatitis virus |
| LDL | low-density lipoprotein |
| TG | triglycerides |
| DG | diacylglycerols |
| FA | fatty acids |
| PC | phosphatidylcholines |
| CE | cholesteryl esters |
| HexCer | hexosylceramides |

**Supplemental Figure 1.** Principal component analysis on 72 significantly differing metabolites between COVID-19 (blue) and the control group (grey). Circles and dots represent the diabetes status.


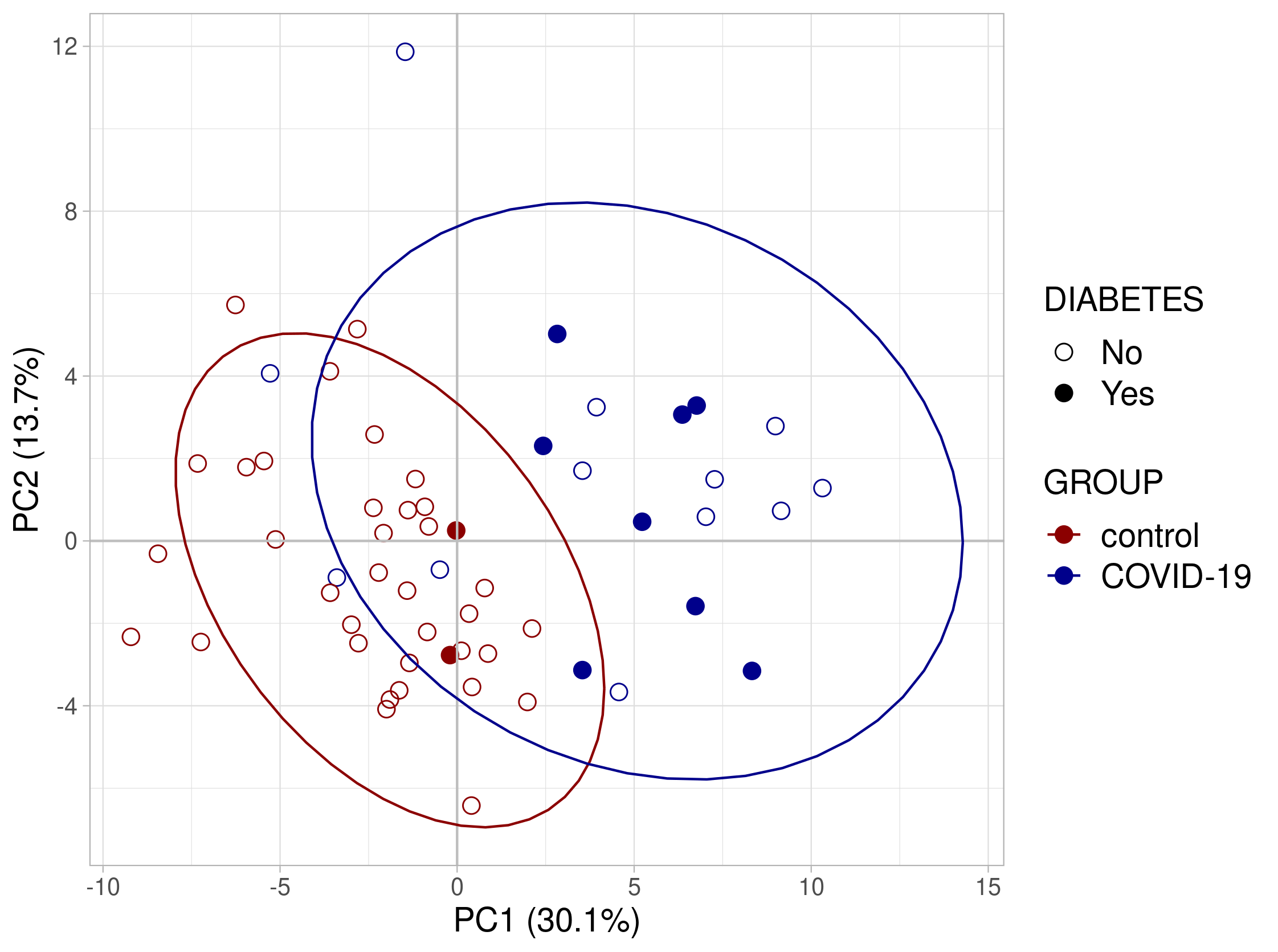


**Supplemental Figure 2.** Receiver-operating curve (ROC) analysis comparing GABA and Cit capacity to discriminate between COVID-19 and control patients. ROC curves were calculated for the absolute quantitative determinations of GABA and Cit in serum. p < 0.05 for GABA, p = 0.067 for Cit. Significance interval on a 98% level, *p* value compared to IL-6. Significance was determined according to de Long et al.

**
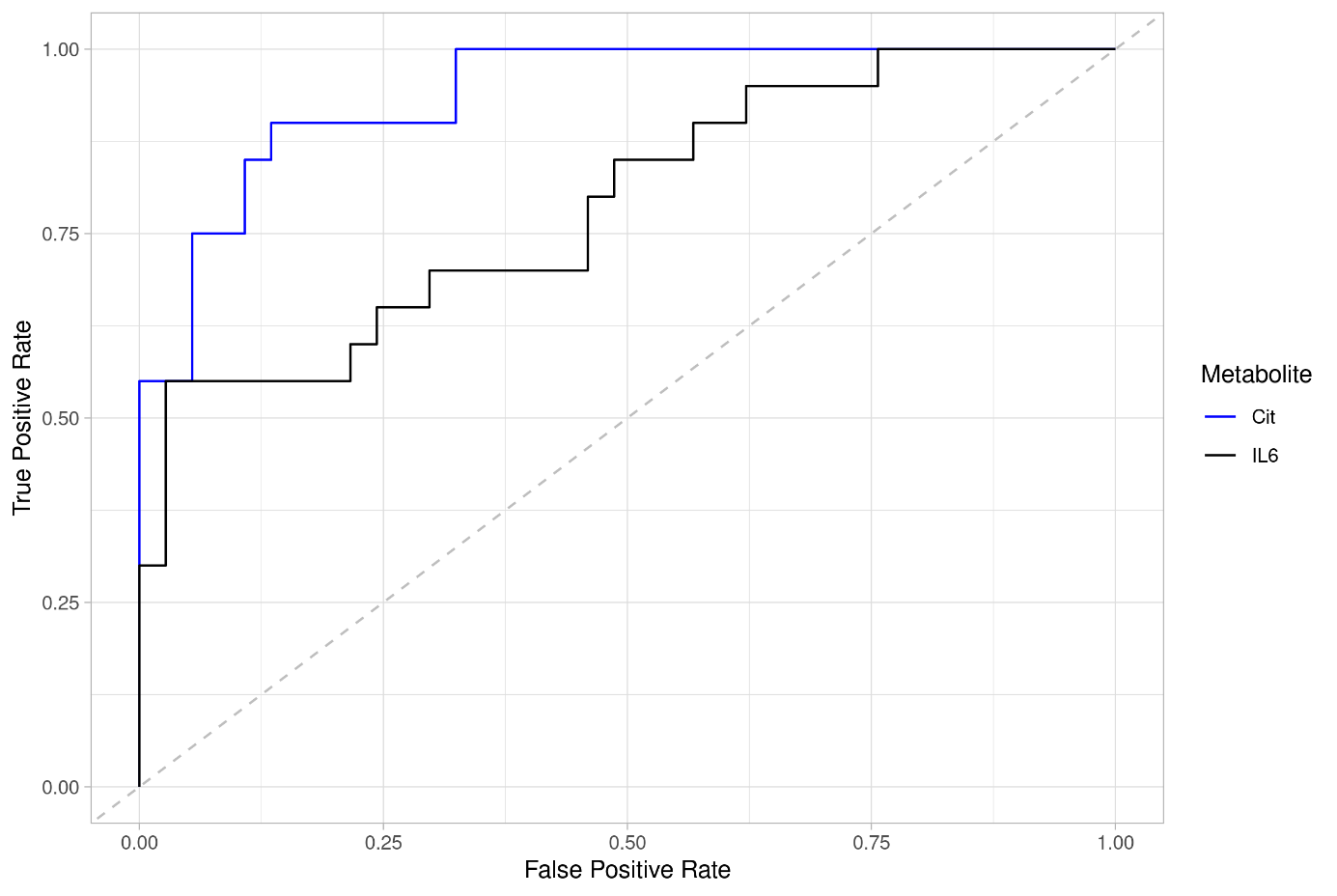
**

**
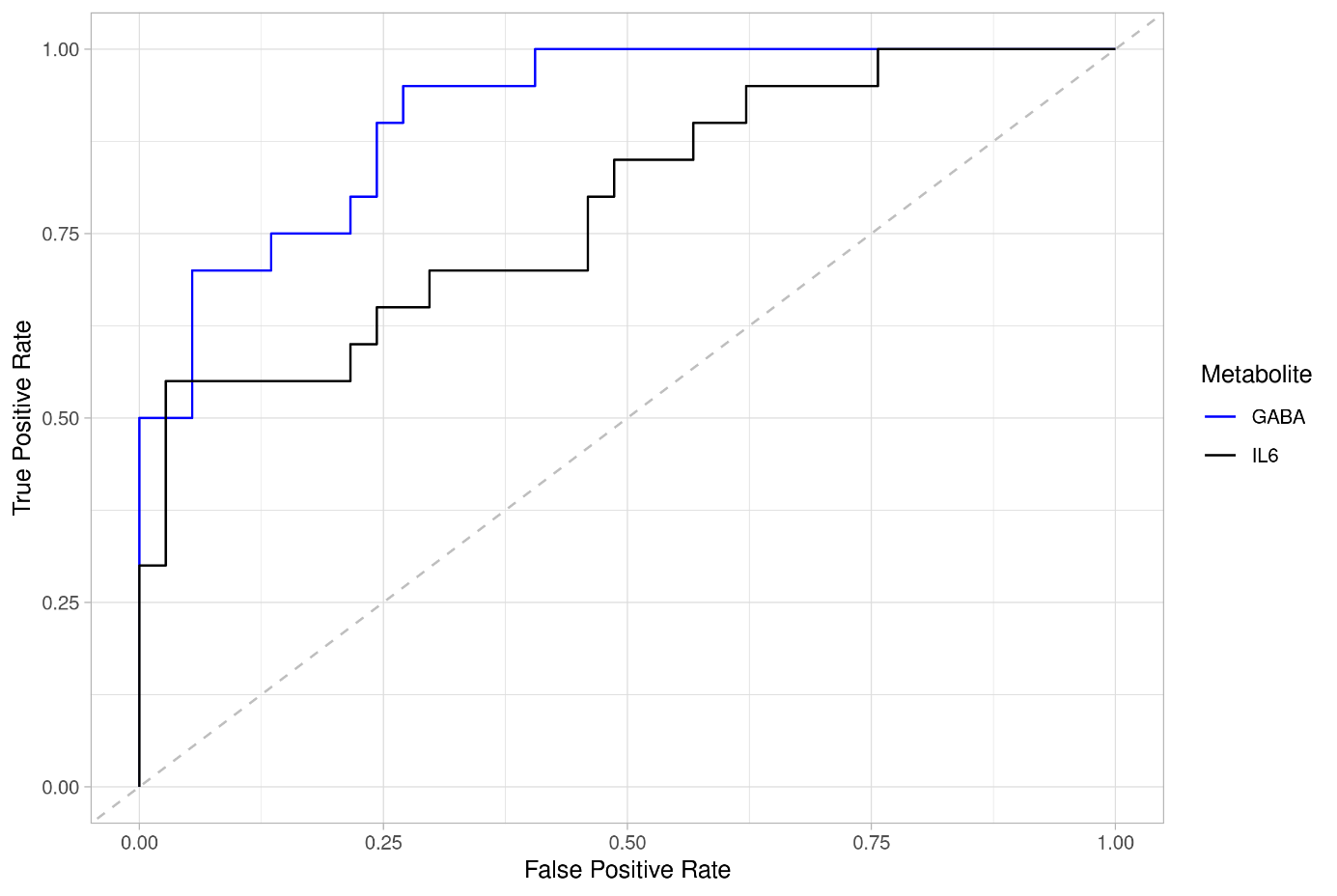
**

**Supplemental Figure 3.** Box-whisker plots showing the distribution of GABA and citrulline in COVID-19 patients with or without diabetes mellitus.


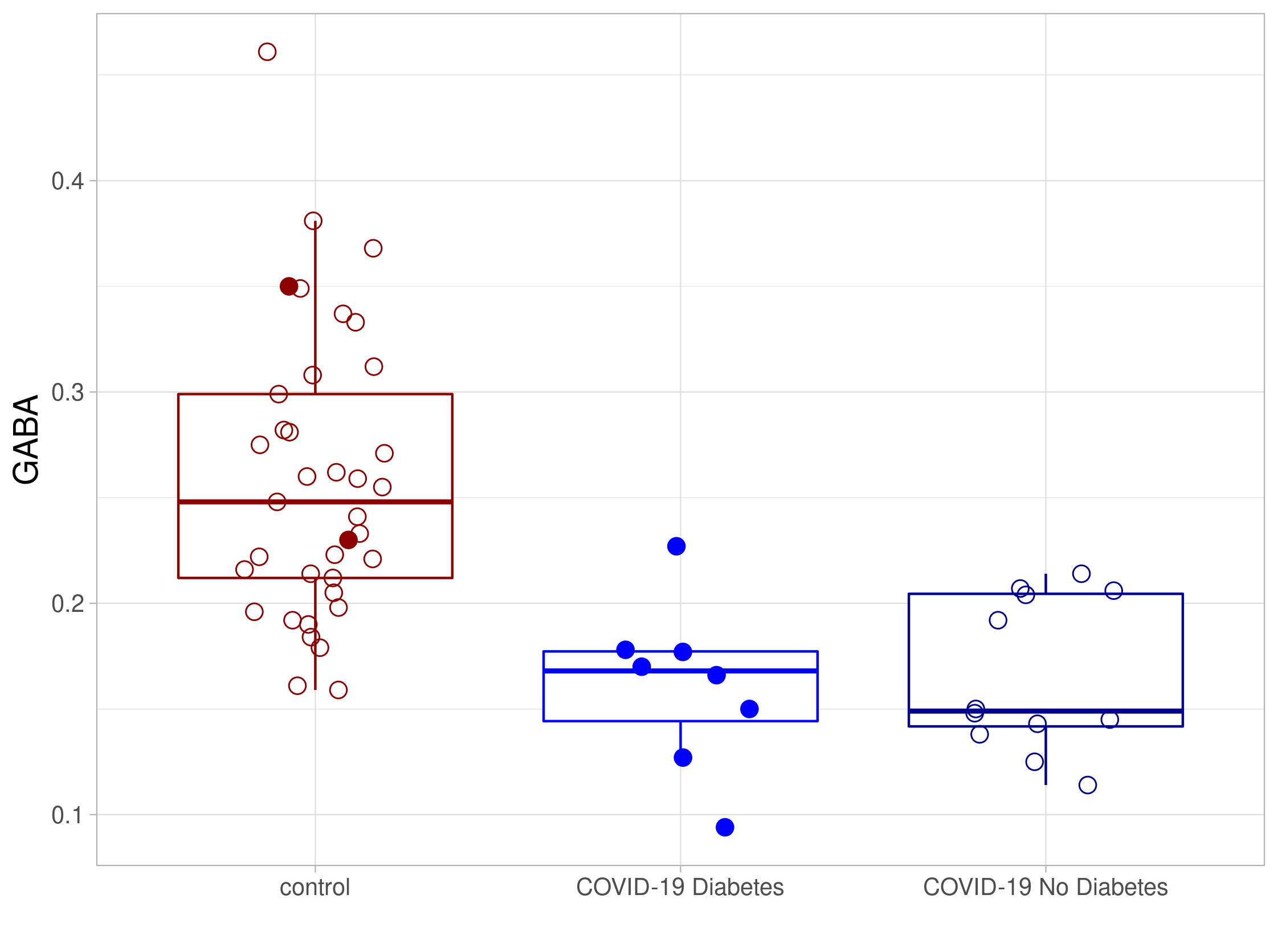

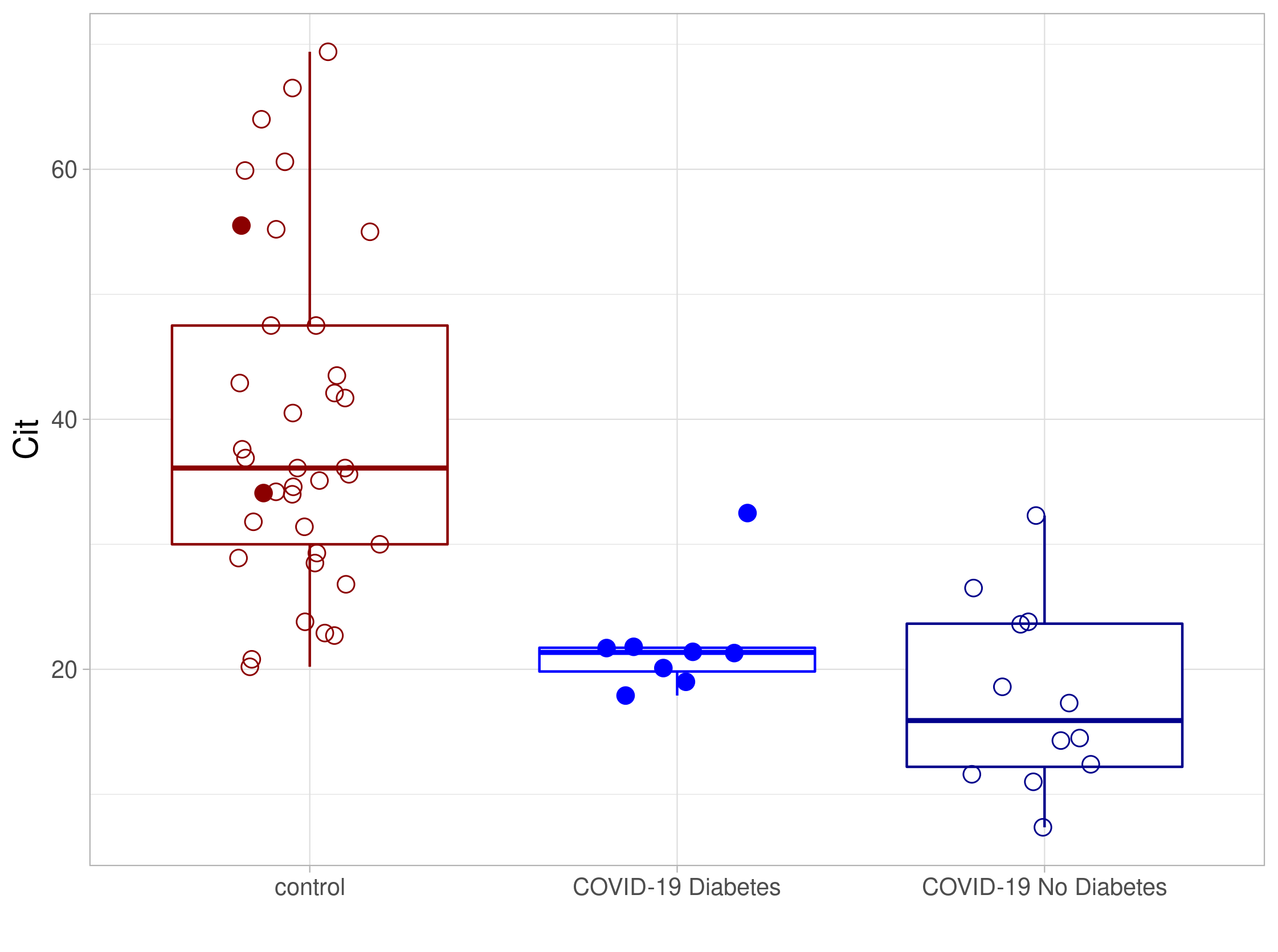

Supplement: Supplementary file 1 — Supplementary file1 (DOCX 748 KB) [file 109_2022_2177_MOESM1_ESM.docx]
